# Diaqua­bis­(4-carb­oxy-2-propyl-1*H*-imidazole-5-carboxyl­ato-κ^2^ *N* ^3^,*O* ^4^)cobalt(II) *N*,*N*-dimethyl­formamide disolvate

**DOI:** 10.1107/S1600536810042054

**Published:** 2010-10-23

**Authors:** Shi-Jie Li, Li-Li Ji, Wen-Dong Song, Shi-Wei Hu, Pei-Wen Qin

**Affiliations:** a College of Food Science and Technology, Guangdong Ocean University, Zhanjiang 524088, People’s Republic of China; b College of Science, Guangdong Ocean University, Zhanjiang 524088, People’s Republic of China; c College of Agriculture, Guang Dong Ocean University, Zhanjiang 524088, People’s Republic of China

## Abstract

In the title complex, [Co(C_8_H_9_N_2_O_4_)_2_(H_2_O)_2_]·2C_3_H_7_NO, the Co^II^ cation (site symmetry 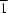) is six-coordinated by two 5-carb­oxy-2-propyl-1*H*-imidazole-4-carboxyl­ate ligands and two water mol­ecules in a distorted octa­hedral environment. In the crystal structure, the complex mol­ecules and dimethyl­formamide solvent mol­ecules are linked by extensive O—H⋯O and N—H⋯O hydrogen bonding into sheets lying parallel to (21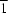).

## Related literature

For our past work based on the 2-propyl-1*H*-imidazole-4,5-carboxyl­ate (H_3_pimda) ligand, see: Yan *et al.* (2010 ▶); Li *et al.* (2010*a* ▶,*b* ▶,*c* ▶,*d* ▶); Song *et al.* (2010 ▶); He *et al.* (2010 ▶); Fan *et al.* (2010 ▶). For Co complexes of a similar ligand, see: Lu *et al.* (2008 ▶); Wang *et al.* (2004 ▶).
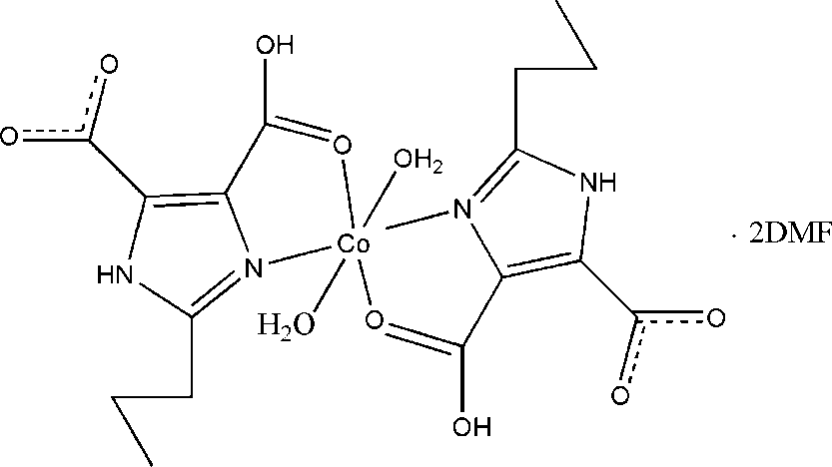


## Experimental

### 

#### Crystal data

[Co(C_8_H_9_N_2_O_4_)_2_(H_2_O)_2_]·2C_3_H_7_NO
*M* *_r_* = 635.50
Triclinic, 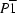

*a* = 7.3325 (7) Å
*b* = 9.330 (1) Å
*c* = 11.2255 (12) Å
α = 76.930 (1)°
β = 87.564 (2)°
γ = 68.857 (1)°
*V* = 697.06 (12) Å^3^
*Z* = 1
Mo *K*α radiation
μ = 0.69 mm^−1^
*T* = 298 K
0.28 × 0.16 × 0.12 mm

#### Data collection

Bruker SMART 1000 CCD area-detector diffractometer
Absorption correction: multi-scan (*SADABS*; Bruker, 2007 ▶) *T*_min_ = 0.831, *T*_max_ = 0.922
3602 measured reflections
2393 independent reflections
1785 reflections with *I* > 2σ(*I*)
*R*_int_ = 0.025

#### Refinement

*R*[*F* ^2^ > 2σ(*F* ^2^)] = 0.046
*wR*(*F* ^2^) = 0.120
*S* = 1.06
2393 reflections
191 parameters
H-atom parameters constrained
Δρ_max_ = 0.37 e Å^−3^
Δρ_min_ = −0.52 e Å^−3^

### 

Data collection: *SMART* (Bruker, 2007 ▶); cell refinement: *SAINT* (Bruker, 2007 ▶); data reduction: *SAINT*; program(s) used to solve structure: *SHELXS97* (Sheldrick, 2008 ▶); program(s) used to refine structure: *SHELXL97* (Sheldrick, 2008 ▶); molecular graphics: *SHELXTL* (Sheldrick, 2008 ▶); software used to prepare material for publication: *SHELXTL*.

## Supplementary Material

Crystal structure: contains datablocks I, global. DOI: 10.1107/S1600536810042054/jh2216sup1.cif

Structure factors: contains datablocks I. DOI: 10.1107/S1600536810042054/jh2216Isup2.hkl

Additional supplementary materials:  crystallographic information; 3D view; checkCIF report

## Figures and Tables

**Table 1:** Hydrogen-bond geometry (Å, °)

*D*—H⋯*A*	*D*—H	H⋯*A*	*D*⋯*A*	*D*—H⋯*A*
O5—H5*D*⋯O4^i^	0.83	2.12	2.946 (3)	174
O5—H5*C*⋯O4^ii^	0.83	1.94	2.773 (3)	175
O2—H2*A*⋯O3	0.82	1.66	2.478 (3)	177
N2—H2⋯O6^iii^	0.86	1.84	2.685 (4)	166

Symmetry codes: (i) 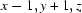; (ii) 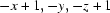; (iii) 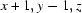.

**Fig. 1.**
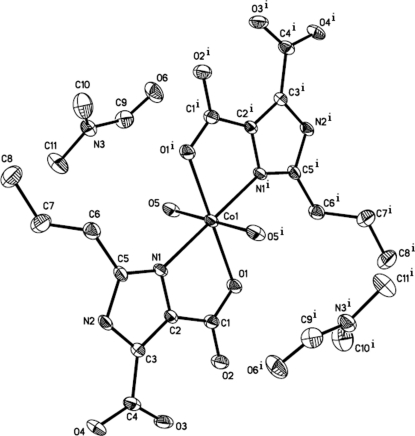
The structure of the title compound, showing the atomic numbering scheme. Non-H atoms are shown with 30% probability displacement ellipsoids. (Symmetry codes: (i)1 - x,1 - y,1 - z;)

**Fig. 2.**
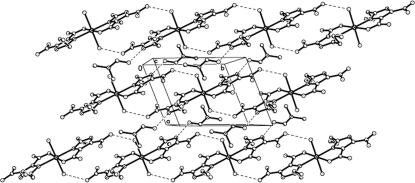
A view of the infinite two-dimensional structure. (H atoms are omitted for clarity)

## supplementary crystallographic information

### Comment

Design of a metal-organic framework *via* deliberate selection of metals and multifunctional ligands is one of the most attractive topics because of the fascinating structural diversity and potential applications in catalysis, chirality, conductivity, luminescence, magnetism, sensors, nonlinear optics, and porosity. 2-propyl-1*H*-imidazole-4,5-carboxylate(H_3_pimda) ligand as one derivative of H_3_IDC with efficient N,*O*-donors has been used to obtain new metal-organic complexes by our research group, such as poly[diaquabis(5-carboxy-2-propyl-1*H*-imidazole-4-carboxylato-k^3^ N^3^, O^4^,*O*^5^)calcium(II)](Song *et al.*, 2010), [diaquabis(5-carboxy-2-propyl-1*H*-imidazole-4-carboxylato-k^2^N^3^,*O*^4^) manganese(II)]*N*,*N*-dimethylformamide(Yan *et al.*, 2010), [Diaquabis(5-carboxy-2-propyl-1*H*-imidazole-4-carboxylato-k^2^*N*^3^,*O*^4^)nickle(II)]*N*,*N*-dimethylformamide disolvate(Li *et al.*, 2010*a*), Diaquabis(4-carboxy-2-propyl-1*H*-imidazole-5-carboxylato- k^2^N^3^,*O*^4^)copper(II) *N*,*N*-dimethylformamide disolvate(He *et al.*, 2010), Diaquabis(5-carboxy-2-propyl-1*H*-imidazole- 4-carboxylato-k^2^N^3^,*O*^4^)nickle(II) tetrahedrate(Fan *et al.*, 2010), Diaquabis(5-carboxy-2-propyl-1*H*-imidazole-4-carboxylato- k^2^N^3^,*O*^4^)-manganese(II) 3.5-hydrate(Li *et al.* 2010*c*), Diaquabis (5-carboxy-2-propyl-1*H*-imidazole-4-carboxylato-K^2^N^3^,*O*^4^)zinc(II) 3.5-hydrate(Li *et al.* 2010*b*), Diaquabis(5-carboxy-2-propyl-1*H*- imidazole-4-carboxylato-k^2^N^3^,*O*^4^)cadmium(II) 3.5-hydrate (Li *et al.* 2010*d*), In this paper, we will report the synthesis and structure of a new Co^II^ complex based the same ligand.

As illustrated in figure 1, the title complex molecule is isomorphous with Ni(II), Mn(II) and Cu(II) analogs (Li *et al.*, 2010a,b,c,d; Yan *et al.*, 2010; He *et al.*, 2010), Similar structural description applies to the present isomorphous complex.the Co^II^ cation lying on the inversion center, is six-coordinated CoN_2_O_4_ in a slightly distorted octahedral geometry, constructed by the two pairs of N and O atoms from H_2_pimda in the equatorial plane, and two coordinate water O atoms occipying the axial position. The Co—O bond lengths and Co—N bond lengths, all of which are within the range of those observed for other Co complexes based on the similar ligand (Lu *et al.*, 2008; Wang *et al.*, 2004). Each H_3_pimda adopts bidentate coordination mode to chelate Co^II^ atom through imidazole N atom and O atom from the protonated carboxyl group, the complex molecules and dimethylformamide solvent molecules are linked by extensive O—H···O and N—H···O hydrogen bonds into a two-dimensional supramolecular network parallel to (001).

### Experimental

A mixture of Co(NO3)_2_ (0.5 mmol, 0.06 g) and 2-propyl-1*H*-imidazole-4,5-dicarboxylic acid(0.5 mmol, 0.99 g) in 15 ml of DMF solution was sealed in an autoclave equipped with a Teflon liner (20 ml) and then heated at 413k for 3 days. Crystals of the title compound were obtained by slow evaporation of the solvent at room temperature.

### Refinement

Water H atoms were located in a difference Fourier map and were allowed to ride on the parent atom, with *U*_iso_(H) = 1.5*U*_eq_(O). Carboxyl H atoms were located in a difference map and refined with distance restraints, *U*_iso_(H) = 1.5*U*_eq_(O). Other H atoms were placed at calculated positions and were treated as riding on parent atoms with C—H = 0.96 (methyl), 0.97 (methylene) and N—H = 0.86 Å, *U*_iso_(H) = 1.2 or 1.5*U*_eq_(C,*N*).

### Crystal data

**Table:**

[Co(C_8_H_9_N_2_O_4_)_2_(H_2_O)_2_]·2C_3_H_7_NO	*Z* = 1
*M**_r_* = 635.50	*F*(000) = 333
Triclinic, *P*1	*D*_x_ = 1.514 Mg m^−^^3^
Hall symbol: -P 1	Mo *K*α radiation, λ = 0.71073 Å
*a* = 7.3325 (7) Å	Cell parameters from 1702 reflections
*b* = 9.330 (1) Å	θ = 2.5–25.9°
*c* = 11.2255 (12) Å	µ = 0.69 mm^−^^1^
α = 76.930 (1)°	*T* = 298 K
β = 87.564 (2)°	Cubic, purple
γ = 68.857 (1)°	0.28 × 0.16 × 0.12 mm
*V* = 697.06 (12) Å^3^	

### Data collection

**Table:**

Bruker SMART 1000 CCD area-detector diffractometer	2393 independent reflections
Radiation source: fine-focus sealed tube	1785 reflections with *I* > 2σ(*I*)
graphite	*R*_int_ = 0.025
φ and ω scans	θ_max_ = 25.0°, θ_min_ = 1.9°
Absorption correction: multi-scan (*SADABS*; Bruker, 2007)	*h* = −8→8
*T*_min_ = 0.831, *T*_max_ = 0.922	*k* = −11→10
3602 measured reflections	*l* = −13→12

### Refinement

**Table:**

Refinement on *F*^2^	Primary atom site location: structure-invariant direct methods
Least-squares matrix: full	Secondary atom site location: difference Fourier map
*R*[*F*^2^ > 2σ(*F*^2^)] = 0.046	Hydrogen site location: inferred from neighbouring sites
*wR*(*F*^2^) = 0.120	H-atom parameters constrained
*S* = 1.06	*w* = 1/[σ^2^(*F*_o_^2^) + (0.0593*P*)^2^ + 0.0702*P*] where *P* = (*F*_o_^2^ + 2*F*_c_^2^)/3
2393 reflections	(Δ/σ)_max_ = 0.001
191 parameters	Δρ_max_ = 0.37 e Å^−^^3^
0 restraints	Δρ_min_ = −0.52 e Å^−^^3^

### Special details

**Table:**

Geometry. All e.s.d.'s (except the e.s.d. in the dihedral angle between two l.s. planes) are estimated using the full covariance matrix. The cell e.s.d.'s are taken into account individually in the estimation of e.s.d.'s in distances, angles and torsion angles; correlations between e.s.d.'s in cell parameters are only used when they are defined by crystal symmetry. An approximate (isotropic) treatment of cell e.s.d.'s is used for estimating e.s.d.'s involving l.s. planes.
Refinement. Refinement of *F*^2^ against ALL reflections. The weighted *R*-factor *wR* and goodness of fit *S* are based on *F*^2^, conventional *R*-factors *R* are based on *F*, with *F* set to zero for negative *F*^2^. The threshold expression of *F*^2^ > σ(*F*^2^) is used only for calculating *R*-factors(gt) *etc*. and is not relevant to the choice of reflections for refinement. *R*-factors based on *F*^2^ are statistically about twice as large as those based on *F*, and *R*- factors based on ALL data will be even larger.

### Fractional atomic coordinates and isotropic or equivalent isotropic displacement parameters (Å^2^)

**Table:**

	*x*	*y*	*z*	*U*_iso_*/*U*_eq_	
Co1	0.5000	0.5000	0.5000	0.0276 (2)	
N1	0.6372 (4)	0.2538 (3)	0.5508 (2)	0.0262 (6)	
N2	0.8031 (4)	−0.0015 (3)	0.6076 (2)	0.0299 (7)	
H2	0.8735	−0.0911	0.6524	0.036*	
N3	0.1217 (5)	0.4898 (4)	0.8633 (3)	0.0431 (8)	
O1	0.4446 (3)	0.4305 (3)	0.3378 (2)	0.0342 (6)	
O2	0.4995 (4)	0.2241 (3)	0.2558 (2)	0.0410 (6)	
H2A	0.5618	0.1289	0.2735	0.061*	
O3	0.6877 (4)	−0.0632 (3)	0.3176 (2)	0.0422 (6)	
O4	0.8630 (4)	−0.2427 (3)	0.4793 (2)	0.0415 (6)	
O5	0.2300 (3)	0.4898 (3)	0.5643 (2)	0.0393 (6)	
H5C	0.2094	0.4117	0.5526	0.047*	
H5D	0.1309	0.5698	0.5403	0.047*	
O6	0.0385 (4)	0.7471 (3)	0.7696 (3)	0.0600 (8)	
C1	0.5195 (5)	0.2858 (4)	0.3436 (3)	0.0300 (8)	
C2	0.6307 (5)	0.1832 (4)	0.4565 (3)	0.0257 (7)	
C3	0.7326 (5)	0.0238 (4)	0.4905 (3)	0.0274 (7)	
C4	0.7665 (5)	−0.1054 (4)	0.4262 (3)	0.0325 (8)	
C5	0.7426 (5)	0.1391 (4)	0.6406 (3)	0.0288 (8)	
C6	0.7851 (6)	0.1544 (4)	0.7649 (3)	0.0380 (9)	
H6A	0.7434	0.2655	0.7652	0.046*	
H6B	0.9254	0.1081	0.7822	0.046*	
C7	0.6851 (7)	0.0760 (5)	0.8653 (3)	0.0535 (11)	
H7A	0.7339	−0.0364	0.8688	0.064*	
H7B	0.5458	0.1168	0.8449	0.064*	
C8	0.7158 (7)	0.1009 (6)	0.9906 (3)	0.0587 (12)	
H8A	0.8492	0.0418	1.0192	0.088*	
H8B	0.6301	0.0656	1.0467	0.088*	
H8C	0.6876	0.2110	0.9853	0.088*	
C9	0.0104 (6)	0.6217 (5)	0.7892 (4)	0.0482 (10)	
H9	−0.0967	0.6196	0.7490	0.058*	
C10	0.2965 (6)	0.4851 (5)	0.9218 (4)	0.0659 (13)	
H10A	0.4092	0.4333	0.8796	0.099*	
H10B	0.3077	0.4281	1.0055	0.099*	
H10C	0.2887	0.5907	0.9190	0.099*	
C11	0.0896 (9)	0.3443 (6)	0.8724 (5)	0.0855 (17)	
H11A	−0.0306	0.3653	0.8290	0.128*	
H11B	0.0817	0.2973	0.9570	0.128*	
H11C	0.1962	0.2733	0.8375	0.128*	

### Atomic displacement parameters (Å^2^)

**Table:**

	*U*^11^	*U*^22^	*U*^33^	*U*^12^	*U*^13^	*U*^23^
Co1	0.0338 (4)	0.0169 (4)	0.0301 (4)	−0.0063 (3)	−0.0011 (3)	−0.0059 (3)
N1	0.0333 (16)	0.0169 (15)	0.0287 (15)	−0.0088 (12)	−0.0018 (12)	−0.0059 (12)
N2	0.0349 (17)	0.0144 (14)	0.0346 (16)	−0.0038 (12)	−0.0030 (13)	−0.0019 (12)
N3	0.049 (2)	0.0271 (18)	0.0475 (19)	−0.0089 (16)	−0.0012 (16)	−0.0055 (15)
O1	0.0437 (15)	0.0207 (13)	0.0315 (13)	−0.0046 (11)	−0.0077 (11)	−0.0025 (10)
O2	0.0544 (18)	0.0285 (14)	0.0357 (14)	−0.0071 (13)	−0.0098 (12)	−0.0098 (12)
O3	0.0545 (17)	0.0317 (15)	0.0424 (16)	−0.0108 (13)	0.0002 (13)	−0.0197 (12)
O4	0.0462 (16)	0.0189 (14)	0.0572 (17)	−0.0063 (12)	−0.0018 (13)	−0.0125 (12)
O5	0.0390 (15)	0.0270 (14)	0.0549 (16)	−0.0118 (12)	0.0054 (12)	−0.0158 (12)
O6	0.066 (2)	0.0279 (16)	0.070 (2)	−0.0047 (14)	−0.0209 (16)	0.0049 (14)
C1	0.033 (2)	0.027 (2)	0.0322 (19)	−0.0115 (16)	0.0004 (15)	−0.0091 (16)
C2	0.0293 (18)	0.0177 (16)	0.0285 (17)	−0.0072 (14)	0.0001 (14)	−0.0043 (14)
C3	0.0319 (19)	0.0228 (18)	0.0295 (18)	−0.0119 (15)	0.0023 (15)	−0.0067 (14)
C4	0.030 (2)	0.025 (2)	0.045 (2)	−0.0092 (16)	0.0079 (17)	−0.0144 (17)
C5	0.034 (2)	0.0179 (18)	0.0322 (19)	−0.0084 (15)	−0.0024 (15)	−0.0016 (15)
C6	0.046 (2)	0.030 (2)	0.036 (2)	−0.0113 (17)	−0.0087 (17)	−0.0054 (16)
C7	0.068 (3)	0.061 (3)	0.040 (2)	−0.029 (2)	0.009 (2)	−0.019 (2)
C8	0.065 (3)	0.068 (3)	0.039 (2)	−0.019 (3)	0.006 (2)	−0.014 (2)
C9	0.043 (2)	0.050 (3)	0.048 (2)	−0.011 (2)	−0.0044 (19)	−0.012 (2)
C10	0.049 (3)	0.055 (3)	0.071 (3)	−0.004 (2)	−0.017 (2)	0.009 (2)
C11	0.126 (5)	0.045 (3)	0.094 (4)	−0.043 (3)	0.017 (4)	−0.016 (3)

### Geometric parameters (Å, °)

**Table:**

Co1—N1^i^	2.098 (3)	O6—C9	1.230 (5)
Co1—N1	2.098 (3)	C1—C2	1.471 (5)
Co1—O5^i^	2.105 (2)	C2—C3	1.372 (4)
Co1—O5	2.105 (2)	C3—C4	1.482 (4)
Co1—O1^i^	2.165 (2)	C5—C6	1.491 (4)
Co1—O1	2.165 (2)	C6—C7	1.513 (5)
N1—C5	1.319 (4)	C6—H6A	0.9700
N1—C2	1.377 (4)	C6—H6B	0.9700
N2—C5	1.357 (4)	C7—C8	1.515 (5)
N2—C3	1.371 (4)	C7—H7A	0.9700
N2—H2	0.8600	C7—H7B	0.9700
N3—C9	1.320 (5)	C8—H8A	0.9600
N3—C11	1.440 (5)	C8—H8B	0.9600
N3—C10	1.447 (5)	C8—H8C	0.9600
O1—C1	1.248 (4)	C9—H9	0.9300
O2—C1	1.286 (4)	C10—H10A	0.9600
O2—H2A	0.8200	C10—H10B	0.9600
O3—C4	1.286 (4)	C10—H10C	0.9600
O4—C4	1.238 (4)	C11—H11A	0.9600
O5—H5C	0.8333	C11—H11B	0.9600
O5—H5D	0.8318	C11—H11C	0.9600
			
N1^i^—Co1—N1	180.0	O4—C4—C3	119.3 (3)
N1^i^—Co1—O5^i^	92.07 (10)	O3—C4—C3	115.5 (3)
N1—Co1—O5^i^	87.93 (10)	N1—C5—N2	110.7 (3)
N1^i^—Co1—O5	87.93 (10)	N1—C5—C6	126.4 (3)
N1—Co1—O5	92.07 (10)	N2—C5—C6	122.8 (3)
O5^i^—Co1—O5	180.0	C5—C6—C7	113.5 (3)
N1^i^—Co1—O1^i^	78.33 (9)	C5—C6—H6A	108.9
N1—Co1—O1^i^	101.67 (9)	C7—C6—H6A	108.9
O5^i^—Co1—O1^i^	88.69 (9)	C5—C6—H6B	108.9
O5—Co1—O1^i^	91.31 (9)	C7—C6—H6B	108.9
N1^i^—Co1—O1	101.67 (9)	H6A—C6—H6B	107.7
N1—Co1—O1	78.33 (9)	C6—C7—C8	113.8 (3)
O5^i^—Co1—O1	91.31 (9)	C6—C7—H7A	108.8
O5—Co1—O1	88.69 (9)	C8—C7—H7A	108.8
O1^i^—Co1—O1	180.0	C6—C7—H7B	108.8
C5—N1—C2	105.8 (3)	C8—C7—H7B	108.8
C5—N1—Co1	142.0 (2)	H7A—C7—H7B	107.7
C2—N1—Co1	111.9 (2)	C7—C8—H8A	109.5
C5—N2—C3	108.3 (3)	C7—C8—H8B	109.5
C5—N2—H2	125.8	H8A—C8—H8B	109.5
C3—N2—H2	125.8	C7—C8—H8C	109.5
C9—N3—C11	121.0 (4)	H8A—C8—H8C	109.5
C9—N3—C10	119.5 (3)	H8B—C8—H8C	109.5
C11—N3—C10	118.7 (4)	O6—C9—N3	124.5 (4)
C1—O1—Co1	114.2 (2)	O6—C9—H9	117.7
C1—O2—H2A	109.5	N3—C9—H9	117.7
Co1—O5—H5C	113.1	N3—C10—H10A	109.5
Co1—O5—H5D	116.9	N3—C10—H10B	109.5
H5C—O5—H5D	108.6	H10A—C10—H10B	109.5
O1—C1—O2	122.4 (3)	N3—C10—H10C	109.5
O1—C1—C2	118.2 (3)	H10A—C10—H10C	109.5
O2—C1—C2	119.5 (3)	H10B—C10—H10C	109.5
C3—C2—N1	110.3 (3)	N3—C11—H11A	109.5
C3—C2—C1	132.5 (3)	N3—C11—H11B	109.5
N1—C2—C1	117.2 (3)	H11A—C11—H11B	109.5
N2—C3—C2	104.9 (3)	N3—C11—H11C	109.5
N2—C3—C4	122.9 (3)	H11A—C11—H11C	109.5
C2—C3—C4	132.2 (3)	H11B—C11—H11C	109.5
O4—C4—O3	125.2 (3)		
			
N1^i^—Co1—N1—C5	156 (25)	O1—C1—C2—N1	2.7 (5)
O5^i^—Co1—N1—C5	85.2 (4)	O2—C1—C2—N1	−175.9 (3)
O5—Co1—N1—C5	−94.8 (4)	C5—N2—C3—C2	0.4 (3)
O1^i^—Co1—N1—C5	−3.0 (4)	C5—N2—C3—C4	−178.4 (3)
O1—Co1—N1—C5	177.0 (4)	N1—C2—C3—N2	−0.5 (3)
N1^i^—Co1—N1—C2	−17 (25)	C1—C2—C3—N2	−179.6 (3)
O5^i^—Co1—N1—C2	−88.1 (2)	N1—C2—C3—C4	178.1 (3)
O5—Co1—N1—C2	91.9 (2)	C1—C2—C3—C4	−1.0 (6)
O1^i^—Co1—N1—C2	−176.3 (2)	N2—C3—C4—O4	−0.3 (5)
O1—Co1—N1—C2	3.7 (2)	C2—C3—C4—O4	−178.6 (3)
N1^i^—Co1—O1—C1	177.5 (2)	N2—C3—C4—O3	178.7 (3)
N1—Co1—O1—C1	−2.5 (2)	C2—C3—C4—O3	0.4 (5)
O5^i^—Co1—O1—C1	85.2 (2)	C2—N1—C5—N2	−0.1 (4)
O5—Co1—O1—C1	−94.8 (2)	Co1—N1—C5—N2	−173.7 (2)
O1^i^—Co1—O1—C1	26 (45)	C2—N1—C5—C6	−177.2 (3)
Co1—O1—C1—O2	179.3 (2)	Co1—N1—C5—C6	9.3 (6)
Co1—O1—C1—C2	0.7 (4)	C3—N2—C5—N1	−0.2 (4)
C5—N1—C2—C3	0.4 (4)	C3—N2—C5—C6	177.1 (3)
Co1—N1—C2—C3	176.1 (2)	N1—C5—C6—C7	110.9 (4)
C5—N1—C2—C1	179.6 (3)	N2—C5—C6—C7	−65.8 (5)
Co1—N1—C2—C1	−4.7 (3)	C5—C6—C7—C8	−175.9 (3)
O1—C1—C2—C3	−178.2 (3)	C11—N3—C9—O6	−174.1 (4)
O2—C1—C2—C3	3.2 (6)	C10—N3—C9—O6	−3.8 (6)

Symmetry codes: (i) −*x*+1, −*y*+1, −*z*+1.

### Hydrogen-bond geometry (Å, °)

**Table:**

*D*—H···*A*	*D*—H	H···*A*	*D*···*A*	*D*—H···*A*
O5—H5D···O4^ii^	0.83	2.12	2.946 (3)	174
O5—H5C···O4^iii^	0.83	1.94	2.773 (3)	175
O2—H2A···O3	0.82	1.66	2.478 (3)	177
N2—H2···O6^iv^	0.86	1.84	2.685 (4)	166

Symmetry codes: (ii) *x*−1, *y*+1, *z*; (iii) −*x*+1, −*y*, −*z*+1; (iv) *x*+1, *y*−1, *z*.
